# Familial Thoracic Aortic Aneurysm and Dissection: Simultaneous Presentation in Two Brothers

**DOI:** 10.7759/cureus.82697

**Published:** 2025-04-21

**Authors:** Kunal Karmilkar, Lis M Llanio, Kyle Maisel, Adesoji Adenigbagbe

**Affiliations:** 1 Internal Medicine, HCA Westside, Plantation, USA; 2 Medical School, Nova Southeastern University Dr. Kiran C. Patel College of Osteopathic Medicine, Davie, USA; 3 Critical Care Medicine, HCA Northwest Hospital, Margate, USA

**Keywords:** abdominal aortic dissection with concurrent aneurysm, aortic dissection, asymptomatic aortic dissection, familial thoracic aortic aneurysm and dissection, htn, thoracic aortic dissection, types of aortic dissection

## Abstract

Familial thoracic aortic aneurysm and dissection (FTAAD) is an inherited condition with variable penetrance, often leading to life-threatening aortic events. We report the cases of two Jamaican brothers who developed acute aortic dissections following the death of a sibling from FTAAD. We reviewed recent literature regarding patient demographics, genetic variability, diagnostic modalities, and management strategies for similar cases. Despite the availability of guidelines, early identification of at-risk individuals and specific management strategies are essential to prevent life-threatening events.

## Introduction

Thoracic aortic aneurysms that lead to acute aortic dissections are a major cause of premature death both in the United States and worldwide. Although physical exam findings, diagnostic imaging, and anti-impulse therapy or surgery are widely established in the diagnosis and management of this condition, over 20% of patients die before arriving at a hospital [1]. Consequently, research has found subtypes of thoracic aortic aneurysm and dissection (TAAD), such as familial TAAD (FTAAD), where genetic predispositions have been found to play a crucial role, along with significant variability in disease onset, severity, and aortic involvement [2]. Given the unpredictable nature of aortic dissections in families with FTAAD, early identification of at-risk individuals and gene-specific management strategies are essential to prevent life-threatening events.

## Case presentation

Case 1

A 50-year-old Jamaican man with a past medical history of uncontrolled hypertension, tobacco and alcohol use disorder, and intracranial hemorrhage (ICH) presented to the emergency room with a one-week history of a progressively worsening headache, nausea, vomiting, and dizziness. He stated that ICH had been diagnosed six months ago due to uncontrolled hypertension. He also reported medication noncompliance, 10 pack-year smoking history, and a family history of FTAAD. Initial vitals were insignificant except for blood pressure of 190/122 mmHg. Physical exam was noncontributory. Routine lab work revealed mildly elevated white blood cells of 13.3 per microliter and an unremarkable complete metabolic panel. CT of the brain demonstrated a right intraparenchymal hemorrhage measuring 1.6 x 2.1 x 1.9 cm involving the caudate, with associated parieto-occipital edema vs. intramural extension. Chest X-ray was negative for acute cardiopulmonary processes, mediastinal widening or loss of aortic knob and EKG showed normal sinus rhythm.

**Figure 1:**
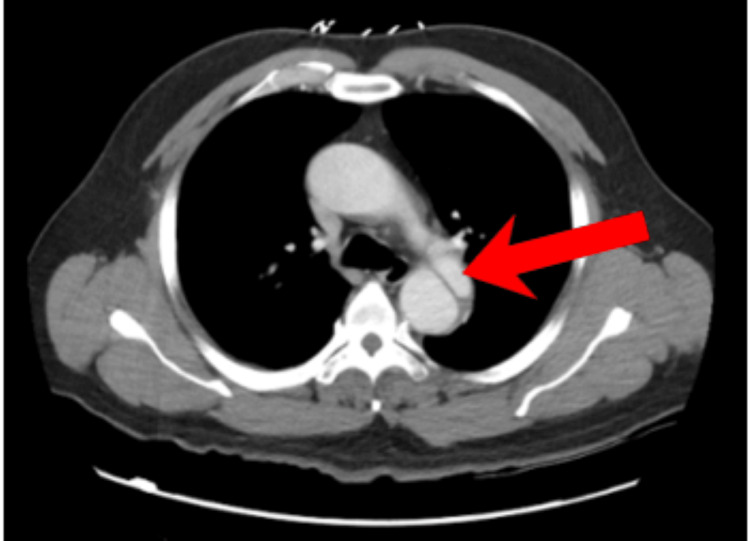
CT chest axial view of aortic dissection (false lumen in descending aorta, red arrow) CT: computed tomography

CT brain findings raised concerns for malignancy due to atypical hemorrhagic features; hence, CT chest, abdomen, and pelvis was performed and revealed no neoplastic appearing lesions, but an incidental aortic dissection (Figure 1). It involved the distal aortic arch measuring approximately 5 cm in length and a 4.9 cm aneurysmal dilatation of the proximal descending thoracic aorta. MRI of the brain demonstrated stable hemorrhagic foci within the right caudate and right parieto-occipital region with stable surrounding vasogenic edema. The patient was started on a nicardipine and esmolol drip for immediate blood pressure control, followed by a transition to oral metoprolol and amlodipine. Despite being offered a thoracic endovascular aortic repair (TEVAR), the patient declined in favor of out-of-state surgical intervention and was discharged with a plan for antihypertensive therapy adjustment. 

Case 2

**Figure 2:**
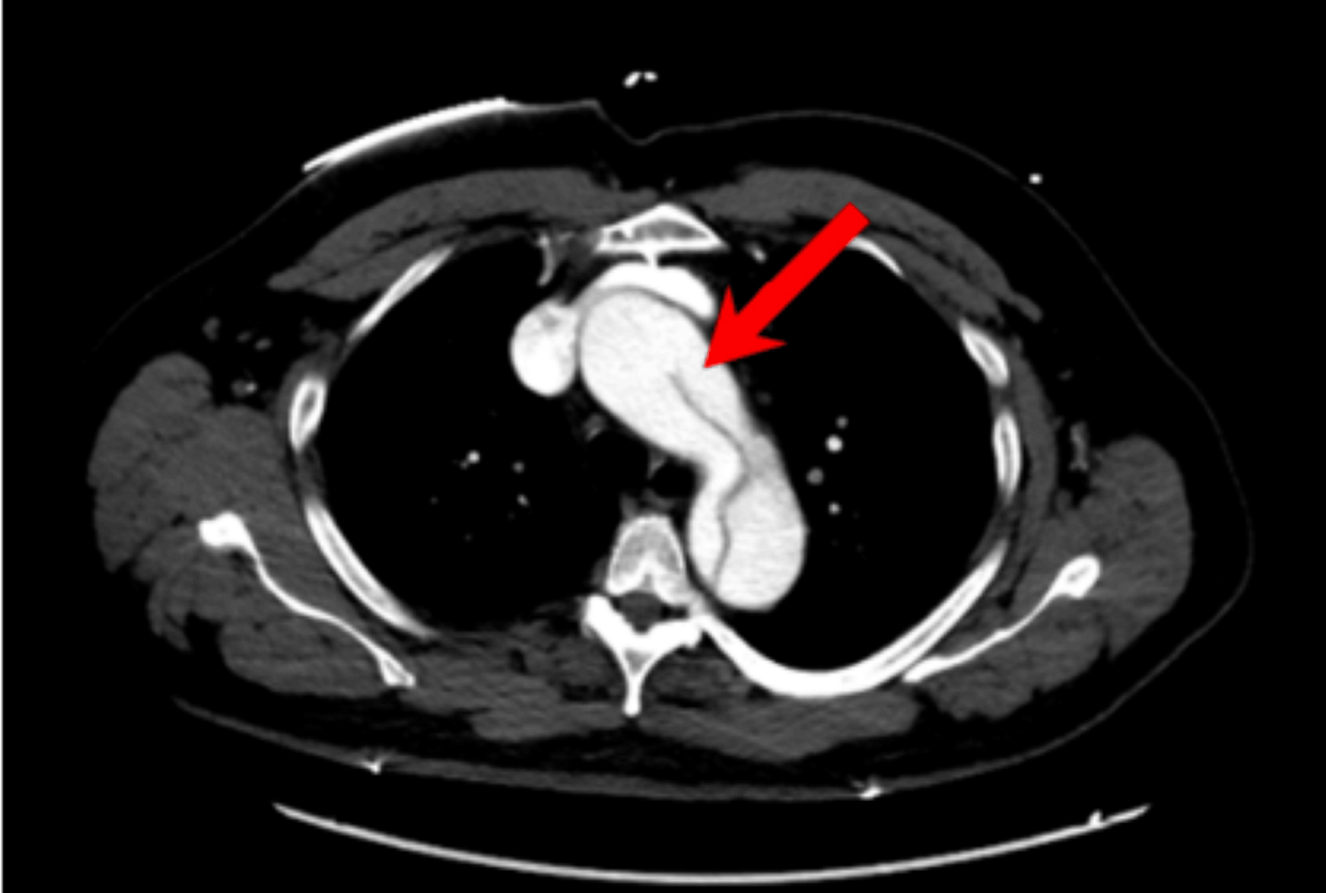
CTA chest axial view of aortic dissection (false lumen in aortic arch, red arrow) CTA: computed tomography angiography

**Figure 3:**
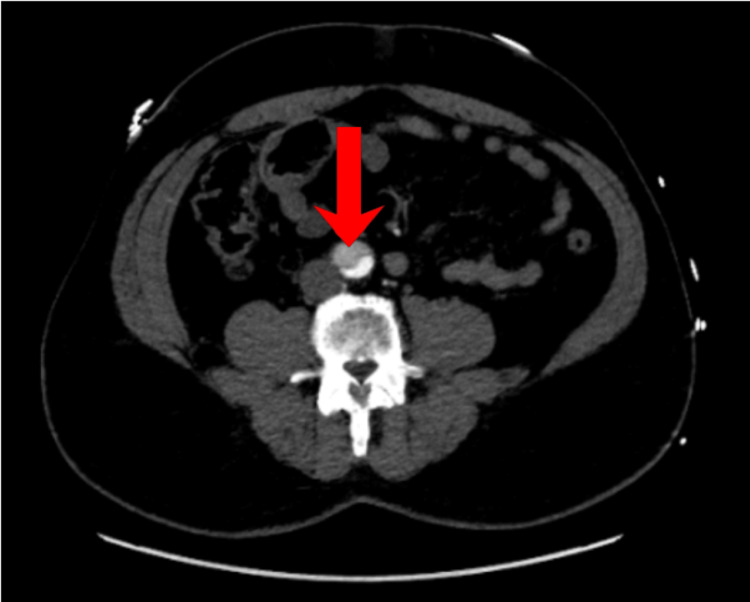
CTA chest axial view of aortic dissection (false lumen in abdominal aorta, red arrow) CTA: computed tomography angiography

A 54-year-old Jamaican man with a past medical history of hypertension, coronary artery disease with stents, and a type A aortic root dissection repaired in 2012 presented to the emergency room with sudden-onset chest pain and associated shortness of breath. He stated that he had begun experiencing a burning chest pain associated with abdominal pain and numbness in the left lower extremity. He denied tobacco, alcohol, or recreational drug use but reported a family medical history of FTAAD. The patient reported that he is amongst seven brothers of whom several had passed from FTAAD, the youngest being 26 years old. Initial vitals were significant only for blood pressure of 160/90 mmHg. The physical exam revealed diminished left lower extremity pulses but was otherwise unremarkable. Labs, including complete metabolic panel and complete blood count, were noncontributory. Chest X-ray revealed cardiomegaly, CT brain was negative, and echocardiogram demonstrated an ejection fraction of 60-65% with moderate dilation of the aortic root, with dissection seen in the proximal ascending aorta. CT angiography (CTA) revealed 4.2 cm dilation of the proximal aortic arch and an aortic dissection beginning in the aortic arch extending to the abdominal aorta (Figures 2, 3). He was immediately started on analgesics, esmolol drip, nicardipine drip, and ultimately underwent an emergent TEVAR with extension to the abdominal aorta.

## Discussion

TAAD is a potentially fatal condition, predominantly occurring as sporadic cases, though nearly 20% are inherited. Its subtypes include syndromic forms, such as Marfan syndrome or Loeys-Dietz syndrome, and nonsyndromic forms, such as FTAAD; however, these conditions exhibit variable penetrance and expressivity [3]. We searched the PubMed database using the keywords “familial aortic dissection” from 2018 to 2024 and applied the filter “free full text” (Table 1). A majority of patients in the 16 case reports identified were male and had unspecified ethnicities. The clinical presentations predominantly included chest pain and dyspnea, with occasional reports of neurological symptoms, fatigue, and palpitations.

**Table 1:** Case reports of FTAAD elicited from the literature review ACTA2: actin alpha cardiac muscle 2; Afib: atrial fibrillation; AI: aortic insufficiency; CT: computed tomography; CTA: computed tomography angiography; FBN1: fibrillin 1; FMH: family medical history; HLD: hyperlipidemia; LDS: Loeys-Dietz syndrome; MHY11: myosin heavy chain 11; MRI: magnetic resonance imaging; MYLK: myosin light chain kinase; RCA: right coronary artery; TEE: transesophageal echocardiogram; TGFBRI: transforming growth factor beta receptor type I; TGFB1: transforming growth factor beta 1; TTE: transthoracic echocardiogram

Study	Patient age, sex, and ethnicity	Presentation	Imaging and findings	Risk factors	Genetics	Management	Outcome
Nishijo et al., 2024 [4]	33, male, Japanese	Chest pain and dyspnea on exertion for months	CTA and TEE; 5.5 cm ascending aorta, dissection from the aortic root to the arch, AI	None	MYLK, FBN1	Surgical repair	Survived
Bobba et al., 2023 [5]	60, male , N/A	Months of intermittent chest pain	CTA and TTE; 4.8 cm aortic root, AI	FMH	ACTA2, R118Q	Surgical repair	Survived
Johnson and Isselbacher, 2023 [6]	68, male, N/A	Asymptomatic	TTE and CTA; 5.1 cm ascending aorta	HTN, HLD, AI, FMH, cardiomyopathy	N/A	Surgical repair	Survived
Ratajska et al., 2022 [7]	46, male, N/A	Dyspnea and fatigue	CT, ascending aorta, and infrarenal abdominal aneurysm	Marfanoid habitus, FMH	SMAD3	Surveillance	Survived
Nickol et al., 2022 [8]	70, male, N/A	Chest pain	CTA and coronary angiogram; 4.2 cm aortic root, 3.9 cm infrarenal AAA, 2.7 cm bilateral iliac artery aneurysm, 0.6 cm RCA aneurysm	LDS, HTN, tobacco use, FMH	TGFBRI	Surgical repair	Deceased
Strecker et al., 2022 [9]	21, male, Caucasian	Chest pain radiating to the back, dyspnea, dizziness, fatigue	TTE, abdominal US, CT; ascending aortic aneurysm and dissection to mesenteric artery level, AI	FMH	ACTA2	Surgical repair	Survived
Chesneau et al., 2021 [10]	46, male, N/A	Sudden-onset chest pain	CT and TTE; 5.0 cm, AI	Prior dissection and repair, FMH	MHY11	Surgical repair	Survived
	45, female, N/A	N/A	CT; aortic root replacement	HTN, tobacco use, FMH	MHY11	Surgical repair	Died from an independent event
Keravnou et al., 2020 [11]	69, female, Cypriot	Dyspnea on exertion, chest pain	CT; aortic root 5.1 cm, ascending aorta 4.7 cm, AI	HLD, varicose veins, intracranial aneurysm, FMH	SMAD3	Surgical repair	Survived, repeat dissection months later
	46, male, Cypriot	Palpitations	CTA and TTE; ascending aorta 6 cm, AI	Marfanoid habitus, paroxysmal Afib, FMH	SMAD3	Surgical repair	Survived
Engström et al., 2020 [12]	59, male, Swedish	Slurred speech, sensory loss in both legs, and neck pain	CT and TTE; 5.3 cm ascending aorta, dissection from the aortic root to the aortoiliac bifurcation, AI	FMH	SMAD3	Surgical repair	Survived, repeat dissection months later
Sathananthan et al., 2020 [13]	28, male, N/A	Pleuritic chest pain radiating down both arms, into the neck, and through the back	Cardiac MRI, TTE, CT; 3.8 cm, dissection from sinotubular junction to brachiocephalic artery, AI	Postpartum, FMH	MYH11	Surgical repair	Survived
Cozijnsen et al., 2019 [14]	61, male, N/A	N/A	CTA, TTE, and MRI; 4.6 cm ascending aorta	FMH	TGFB1	Surgical repair	Survived
Kane and Shamsa, 2019 [15]	21, male, N/A	N/A	CTA, TTE; 3.7 cm aortic root	FMH	SMAD3	Surveillance	Survived
Keravnou et al., 2018 [16]	30, male, Cypriot	Chest and back pain	TTE and CT; 7.0 cm aortic root with dissection sinotubular junction to aortic root, AI	FMH	ACTA2, MYH11	Surgical repair	Survived
Erhart et al., 2018 [17]	36, male, N/A	Ischemic stroke	Imaging N/A; dissection extending into both carotid arteries	FMH	16p13.1 duplication	Surgical repair	Survived

Family medical history was reported in 94% of cases, with hypertension, hyperlipidemia, marfanoid habitus, and tobacco use being the most common additional risk factors. CTA and transthoracic echocardiogram (TTE) were the most frequently utilized imaging modalities to confirm diagnosis and prognosticate the dissection. Approximately 75% of cases involved aneurysms of the ascending aorta and aortic root, with an average of 4.93 cm. Aortic dissection was present in over 50% of cases, and its extent continuously varied. However, aortic insufficiency was also present in 62% of cases, highlighting its association with FTAAD. The most common genetic mutations identified were SMAD3, followed by ACTA2 and MYH11, with less frequent mutations including MYLK, FBN1, TGFBRI, TGFB1, and chromosomal 16p13.1 duplication. Of the cohort, 75% underwent surgical repair while 25% were managed conservatively. The survival rate was 93%, with only one patient passing from FTAAD itself.

Unfortunately, our patients presented shortly after attending the funeral of their elder sibling, who had recently passed away in his early 50s from a FTAAD. This raised concerns about emotional stress acting as a trigger for aortic events in those with genetic susceptibility. Research shows that stress can elevate blood pressure to hypertensive crisis levels (180/110), a phenomenon known as blood pressure reactivity [11]. We hypothesize that this sudden increase in pressure could explain the simultaneous dissections in our patients. While stress management was not directly discussed in any of the case reports, research has shown it can precipitate FTAAD due to transient, severe hypertension [18]. Although not performed during their hospitalization, we strongly recommended genetic testing to assess hereditary risks and initiate early screening for their children. Current American College of Cardiology (ACC) and American Heart Association (AHA) guidelines do not provide specific recommendations on the age to begin screening individuals with a family history of FTAAD [19]. We recommend initiating screening with TTE or CTA at age 18 or 10 years before the earliest known diagnosis of aortic disease within the family, whichever comes first, to enable timely detection and intervention. 

Our study is limited by its small sample size and reliance on case reports alone, thereby reducing its generalizability. Nonetheless, the variability in clinical presentations, genetic testing, and management underscores the need for standardized protocols. Future research should involve larger cohorts to define an optimal screening timeframe and integrate lifestyle modifications, including stress management, to improve patient outcomes.

## Conclusions

This report highlights the need for early screening and management of FTAAD. Current guidelines do not provide clear recommendations for screening timelines in individuals with a family history or include stress management as part of lifestyle modifications, underlining an important gap in clinical practice.
